# Successful treatment of anterior segment fibrosis with intravitreal methotrexate

**DOI:** 10.1016/j.ajoc.2021.101247

**Published:** 2021-12-31

**Authors:** Tessnim R. Ahmad, Ying Han, Jay M. Stewart

**Affiliations:** a University of California, San Francisco, Department of Ophthalmology, San Francisco, CA, USA; b Zuckerberg San Francisco General Hospital and Trauma Center, Department of Ophthalmology, San Francisco, CA, USA

**Keywords:** Fibrosis, Methotrexate, Uveitis, Glaucoma, Membranectomy

## 1 Case report

A 20-year-old man presented to an outside ophthalmologist for vision loss over four weeks. Vision was hand motion in the right and light perception in the left. Both eyes had trace white blood cells (WBC), marked flare, white cataracts, and retinal detachments. The patient was treated for panuveitis and underwent cataract extraction and posterior chamber intraocular lens placement (CE/IOL) in the left eye.

Upon our first examination at postoperative month 3, the left eye had persistent inflammation, a pupillary IOL membrane, and iridocorneal touch nasally and superiorly. Vision was 20/250 and intraocular pressure (IOP) was 26 mm Hg. Over the following two months, the eye developed near-complete iridocorneal touch and anterior IOL displacement. IOP reached 55 mm Hg despite maximal topical and oral medications. The patient underwent Ahmed glaucoma valve (AGV) placement and membranectomy.

**Fig. 1:**
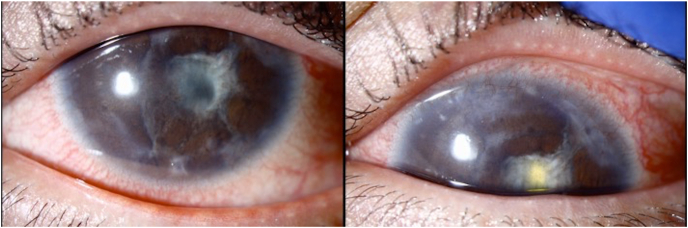
Extensive fibrosis across the pupil, anterior chamber, and AGV tube 1 month after Ahmed glaucoma valve (AGV) placement and membranectomy.

**Fig. 2:**
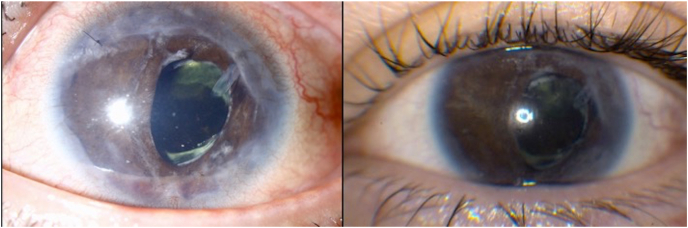
Clear anterior segments 1 month (left) and 4 months (right) after membranectomy with methotrexate.

At postoperative month 1 after AGV implantation, vision was 20/100, IOP was 10 mm Hg, and there was moderate flare. Extensive membranes across the pupil, anterior chamber, and AGV tube had formed (Fig. 1). At postoperative month 5, vision was counting fingers. The patient underwent repeat membranectomy with intraoperative and then weekly postoperative intravitreal methotrexate (400 mcg/0.1 mL; off-label) for a total of eight doses. The anterior chamber was clear during this two-month period and for three months thereafter (Fig. 2). The patient was lost to follow-up for one month. Re-examination demonstrated a quiet eye with few membranes in the temporal anterior chamber and AGV tube. Repeat membranectomy was performed followed by a second series of intravitreal methotrexate. Pathology demonstrated dense connective tissue with pigment deposition and no inflammation. Monthly methotrexate is planned.

## 2 Discussion

Anterior segment fibrosis in this patient may be multifactorial. The initial fibrosis may be related to chronic inflammation due to uveitis and/or surgery. He may have further developed a non-inflammatory fibrosis with a mechanism similar to that reported in eyes with iris defects.^1^ Methotrexate has recently shown promise for treating proliferative vitreoretinopathy (PVR)^2^ and epithelial ingrowth.^3^ This report suggests its efficacy for anterior segment fibrosis when given in a dosing regimen similar to that for PVR. The mechanism may be direct inhibition of fibrous proliferation and/or inflammation. Maintenance therapy may be required.

## 3 Conclusion

This is the first report to suggest the efficacy of intravitreal methotrexate for anterior segment fibrosis. Further investigation to establish methotrexate's efficacy (as a surgical adjunct or primary intervention) and optimal dosing is warranted.

## Patient consent

Written consent to publish this case has not been obtained. This report does not contain any personal identifying information.

Acknowledgements and Disclosures.

## Funding

Support from That Man May See, Inc., and Research to Prevent Blindness.

## Authorship

All authors attest that they meet the current ICMJE criteria for authorship.

## Declaration of competing interest

The authors have no conflicts of interest.
